# Psychometric properties of the Activities Scale for Kids-performance after allogeneic hematopoietic stem cell transplantation in adolescents and children: Results of a prospective study on behalf of the German-Austrian-Swiss GVHD Consortium

**DOI:** 10.1007/s00508-020-01641-w

**Published:** 2020-04-03

**Authors:** Anita Lawitschka, Matthias Brunmair, Dorothea Bauer, Natalia Zubarovskaya, Rosemarie Felder-Puig, Brigitte Strahm, Peter Bader, Gabriele Strauss, Michael Albert, Irene von Luettichau, Hildegard Greinix, Daniel Wolff, Christina Peters

**Affiliations:** 1 St. Anna Children’s Hospital, SCT-Outpatient & Aftercare Clinic, Medical University Vienna and Children’s Cancer Research Institute, Kinderspitalgasse 15, 1090 Vienna, Austria; 2 Department of Psychology, University of Wuerzburg, Wuerzburg, Germany; 3 Ludwig Boltzmann Institute Health Technology Assessment, Vienna, Austria; 4 Department of Paediatrics and Adolescent Medicine, Division of Paediatric Haematology and Oncology, Medical Centre, University of Freiburg, Freiburg, Germany; 5 Division for Stem Cell Transplantation, University Children’s Hospital, Frankfurt/Main, Germany; 6 Department for Paediatric Oncology and Haematology, HELIOS Klinikum Berlin-Buch, Berlin, Germany; 7 Department of Pediatrics, Division of Pediatric Haematology and Oncology, Dr von Hauner Children’s Hospital, LMU, Munich, Germany; 8 Children’s Hospital Medical Centre, Technical University of Munich, Munich, Germany; 9 Division of Hematology, Medical University of Graz, Graz, Austria; 10 Department of Internal Medicine III, University Hospital Regensburg, F. J. Strauss Allee 11, 93053 Regensburg, Germany

**Keywords:** Physical functioning, Cancer patients, AYAs, GVHD

## Abstract

**Background:**

The psychometric properties of an instrument, the Activity Scale for Kids-performance (ASKp), were assessed which was proposed to capture physical functioning after allogeneic hematopoietic stem cell transplantation (HSCT). Additionally, this multicenter observational prospective study investigated the influence of clinical correlates focusing on chronic graft-versus-host disease (cGVHD).

**Methods:**

Patient-reported ASKp, clinician-reported Karnofsky/Lansky status (KPS/PSS), patient characteristics and cGVHD details were assessed of 55 patients with a median age of 12 years at baseline after day +100 post-HSCT and every 3 months during the next 18 months. The psychometric properties were evaluated and ASKp and KPS/PSS status was compared using ANOVAS and multiple regression models.

**Results:**

The German version of the ASKp showed good psychometric properties except for ceiling effects. Discrimination ability of the ASKp was good regarding the need for devices but failed to predict cGVHD patients. Both the ASKp and the KPS/PSS were associated with patients after adoptive cell therapy being in need for devices, suffering from overlap cGVHD and from steroid side effects but not with patients’ age and gender. In contrast to the KPS/PSS the ASKp only showed significant differences after merging moderate and severe cGHVD patients when comparing them to No-cGVHD (F = 4.050; *p* = 0.049), being outperformed by the KPS/PSS (F = 20.082; *p* < 0.001).

**Conclusion:**

The ASKp showed no clear advantages compared to KPS/PSS even though economical and patients’ effort was higher. Further application range may be limited through ceiling effects. Both should be taken into consideration. Therefore, the results may not support the usage of ASKp after HSCT and rather suggest KPS/PSS, both patient and clinician reported.

## Introduction

Allogeneic hematopoietic stem cell transplant (HSCT) offers a curative treatment for advanced malignancies and otherwise incurable diseases of hematologic or immunologic origin. The increasing number of HSCT survivors are subject to many comorbidities [1–3] among which the most significant affecting survival and multidimensional aspects of quality of life (QOL) are acute (aGVHD) and chronic graft-versus-host disease (cGVHD) [4, 5]. Chronic GVHD is an extremely complex multisystemic chronic disorder with heterogeneous pathogenesis [6] and clinical manifestations resembling a variety of autoimmune diseases with involvement of many tissues and organs in adults, adolescent and pediatric patients [7, 8]. Furthermore, cGVHD is associated with decreased survival, prolonged duration of immunosuppression [9], adverse physical functioning, disability [10] and decreased QOL [11, 12].

Physical functioning is of major importance for the long-term outcome after HSCT including various aspects, such as daily activity, fatigue, distress, QOL, social role and reintegration into the community. Generally, patients experience considerable physical and functional deterioration during and after HSCT. Physical activity and exercise have been shown to clearly enhance fitness, improve symptoms of sequelae and QOL both in pediatric and adult survivors. Over the last years, the number of reports and recommendations, which support the assessment of physical functioning and the implementation of physical exercise as part of the supportive care regimens for cancer and HSCT patients have increased [10, 17].

Diagnosis of cGVHD is difficult and identification of accurate standardized instruments to measure cGVHD outcomes is very important. In 2005, the National Institutes of Health (NIH) consensus development project on criteria for clinical trials in cGvHD established standardized definitions for diagnosis, severity scoring and response measures [6, 13].

A review by Krupski e al. [14] summarized the currently available trials that have shown the association of NIH-defined overall severity of cGVHD (mild, moderate and severe) and/or clinical response to cGVHD treatment with various outcome measures and QOL. Additionally, in 2005 the NIH consensus characterized overlap cGVHD, a subtype with symptoms both of acute and cGVHD, which is associated with some degree of worse outcome [11, 15].

The association between physical function and QOL in patients with cGVHD has clearly been shown outlining the importance of collecting patient reported measures in prospective studies [11, 16, 17]. In this respect, the proposed instruments of the 2005 NIH consensus [6, 13] include the human activity profile (HAP) [16] for adults and the activity scale for kids-performance (ASKp) for children [18]. The latter is a self-report measure of childhood physical disability that has shown excellent reliability in children with musculoskeletal limitations [19]. The HAP has proved to be associated with global severity, overlap subtype of cGVHD and with change in cGVHD status [11, 16].

Performance scales, such as the Karnofsky performance scale (KPS) [20] for adolescents and adults over 16 years of age, and the Lansky play performance scale (PSS) for children below 16 years of age [21] are also in use to assess functional status after HSCT [22–24]. Some studies have shown a negative correlation with global severity of cGVHD and with joint and fascial involvement of cGVHD [25, 26].

To our knowledge, no study has examined the performance of the ASKp in pediatric HSCT patients so far. The aim of this project was to fill this gap and to determine the psychometric properties of a German-language version of the ASKp.

## Patients and methods

### Patients

A German version of the ASKp was evaluated in pediatric HSCT survivors in a multicenter observational prospective study in four German HSCT centers and one Austrian center. Written informed consent in accordance with the Declaration of Helsinki and the institutional review board of the Medical University of Vienna and the St. Anna Children’s Hospital was obtained. Furthermore, the project was reviewed and approved by the institutional ethics board at each center. Patients were enrolled during regular visits at the HSCT centers. All patients and parents were informed that they were not given the individual results of the project evaluations. Inclusion criteria were a life expectancy of more than 3 months, at least 2 years of age and no evidence of recurrence of primary disease. The assessments were performed after day +100 every 3 months for 18 months (i.e. T‑0, 3, 6, 9, 12, 15 and 18 months) and when clinically indicated. A physician routinely documented patient and transplant characteristics at study entry; In addition, at all study time points the i) clinical status including KPS/PSS, relevant comorbidities such as the need for devices, gastrointestinal (GI, like nausea and malabsorption) and endocrine comorbidity (such as diabetes, hypothyroidism, adrenal dysfunction, hypogonadism), ii) corticosteroid side effects (such as Cushing syndrome and myopathy) and iii) according to the NIH consensus criteria on cGVHD global severity (mild-moderate-severe) and cGVHD severity assessment (10-point scale) [6, 13]. Patient cohorts were grouped into no cGVHD and classical cGVHD; the latter only incorporated patients with moderate and severe cGVHD as there were too few patients with mild cGVHD to analyze. Additionally, classical cGVHD included overlap subtype, presenting symptoms both of acute and cGVHD. Late acute or recurrent acute GVHD was excluded from the analyses. In accordance with the NIH criteria cGVHD-associated symptoms (cGVHD-ASS, such as eosinophilia, thrombocytopenia, (poly)serositis, nephrotic syndrome, myasthenia gravis, peripheral neuropathy) [6] were also evaluated at the time of testing. The need of help in answering the questionnaire at time of testing was categorized using a 4-point scale (1 = no help needed, 2 = questions were read to the child, 3 = help with some questions, 4 = help with most of the questions).

### Activity Scale for Kids-performance (ASKp)

**Fig. 1:**
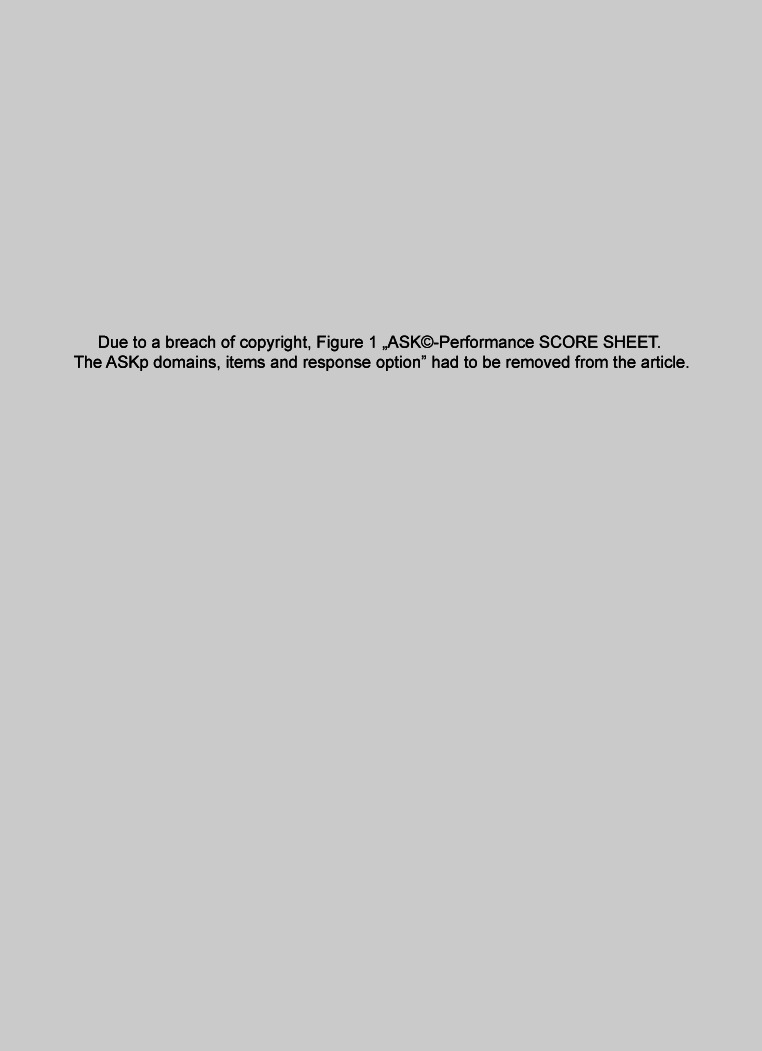
ASK©-Performance SCORE SHEET. The ASKp domains, items and response options

The ASKp is a self-reported measure of childhood physical function and physical disability, which has excellent reliability and validity [18]. The ASKp contains 30 items which are rated from 0 = never to 4 = always and covers 9 different domains. It has shown excellent measurement properties in children with musculoskeletal limitations [19] but has not been tested in HSCT patients. The ASKp domains, items and response options are displayed in Fig. 1. Possible summary scores of the ASKp range from 0 to 120 points.

### Biostatistical methods

All statistical analyses of data accuracy, measurement properties, descriptive statistics, multiple regressions and discrimination analysis were conducted with the statistical package for Social Sciences 22.0 (SPSS Inc., Chicago, IL, USA). Corresponding assumptions for the statistical procedures were checked. The Wilcoxon sign rank test was used to analyze changes over time. Furthermore, we used aggregated data to test our other hypothesis.

Two main issues emerged out of different sample structure at each time point. First, sample composition was different at the various measurement time points. Second, aggregated individual ASKp values were affected by differences between time points. To control this, we created two different mean values across time points: we computed the ASKp mean for each patient across all time points. These mean values are not affected by different sample compositions, but by convalescence, training and other differences between time points. To control convalescence and other differences we standardized patient values at every time point by the sample mean and standard deviation at this time point. Then we computed standardized mean values across all time points. In contrast to the unstandardized mean the standardized value is not affected by time point differences but by different sample compositions at the time points. We analyzed the performance of the ASKp to predict the need of devices to measure validity. Thereby we used the receiver operating characteristic curve. The area under the curve (AUC) summarizes the power of the prediction model. The AUC values are between 0 and 1. Higher AUC values than 0.5 indicate a better prediction than expected by change and lower values vice versa. To examine the predictive value of ASKp scores and the associations of the ASKp with cGVHD we used multiple regression techniques.

## Results

Of 92 HSCT study patients the ASKp was completed by 71 patients at least at 1 time point, with a complete data set of 55 patients with a median age of 12 years (range 4–23 years). Of the patients 64% were male. The underlying diseases as indication for HSCT were malignant in 78% (non-malignant in 22%) and detailed patient and HSCT characteristics at study enrolment are displayed in Table 1. Including the one patient with mild cGVHD, the no-cGVHD group consisted of 35 patients. The cohort of cGVHD patients (19) included classic chronic GVHD in 9/19 and overlap cGVHD in 10/19 patients. The NIH overall severity was moderate in 11 (58%) and severe in 7 (37%) patients. Most commonly involved organs were skin, mouth and eyes. Of note, 9/19 (47%) of cGVHD patients suffered from joint involvement. The onset type was quiescent in 6/19, progressive in 9/19 and de novo in 4/19 patients. Of the 19 cGVHD patients 17 (90%) had a history of prior aGVHD.

**Table 1:** Patient and HSCT characteristics at study enrolment

	*N*	%		*N*	%
Total	55	100	Total	55	100
Gender	–	–	Donor	–	–
Female	20	36	MUD	21	38
Male	35	64	MFD	23	42
Age in years	–	–	MMD	11	20
*Mdn*, range (years)	12.0	4–23	Adoptive cell therapy	8	14
Underlying diseases	–	–	No-cGVHD	36	65
Malignant diseases	43	78	cGVHD	19	35
Acute leukemia, MDS	32	58	Classic	9/19	47
Solid tumors, lymphomas	11	20	Overlap	10/19	53
Non-malignant diseases	12	22	Mild	1/19	5
Hemoglobinopathies, BM failures, MLD	9	16	Moderate	11/19	58
Immunodeficiencies	3	6	Severe	7/19	37
Conditioning regimens	–	–	Onset type	–	–
MAC	41	75	Quiescent	6/19	32
RIC	14	25	Progressive	9/19	47
TBI	23	42	De novo	4/19	21
Stem cell source	–	–	Prior aGVHD	17/19	90
BM	41	75	cGVHD after adoptive cell therapy	5/19	26
PB	14	25	Joint involvement	9/19	47

*BM* bone marrow, *aGVHD* acute graft-versus-host disease, *cGVHD* chronic graft-versus-host disease, *CML* chronic myeloid leukemia, *ID* immune deficiencies, *Mdn.* median, *MAC* myeloablative conditioning, *MDS* myelodysplastic syndromes, *MFD* matched family donor, *MLD* metachromatic leukodystrophy, *MMD* mismatched donor, *MUD* matched unrelated donor, *PB* peripheral blood stem cells, *RIC* reduced intensity conditioning, *TBI* total body irradiation

During the study period the ratio of patients with cGVHD to those without (no-cGVHD) was about 1:3 until time point T9 and 1:4 later. Overall, severe cGVHD was evident at 38 study points, moderate in 22 and mild cGVHD in 11 study points.

Of 8 patients with a history of adoptive cell therapy (donor-lymphocyte infusion 2/8, mesenchymal stem cells 2/8, donor-lymphocyte infusions plus mesenchymal stem cells 1/8, stem cell boost 2/8, virus-specific T cells 1/8 patients) at study enrolment, 4 patients experienced moderate or severe cGVHD (50%), all of them classified as the subtype of overlap cGVHD and 3/4 experiencing steroid side effects. Of 47 patients without adoptive cell therapy, 15 patients suffered from moderate or severe cGVHD (32%), 6 of these 15 patients presented with an overlap subtype of cGVHD and 5/15 with steroid side effects.

### Item characteristics and ceiling effects

The item “I took care of my medical needs” was excluded from all further analyses because weighted mean corrected item-scale correlation across time points was low (*r*_it_ = 0.18) and the reliability of the ASKp scale was higher without this item. All other items showed good item characteristics with weighted mean corrected item-scale correlations of *r*_it_ = 0.44 to *r*_it_ = 0.74. Ceiling effects increased with time: 1 patient gave the highest possible rating of 130 points at T0 (2%), 3 patients at T3 (7%) and T6 (9%), 6 patients at T9 (23%), 9 patients at T12 (35%) and T18 (33%) and 5 patients at T15 (22%).

### Reliability

Internal consistency and retest-reliability values of the ASKp for different time points and time lags are shown in Table 2. The internal consistency was above average at all time points with Cronbach’s alpha ranging from α = 0.91 to α = 0.97. Moreover, the intraclass correlation coefficient was good (ICC = 0.90) and even better when excluding T9 results (ICC = 0.96). Furthermore, we compared re-test reliability for every single time point and every single time lag. Associations were low for T0 and T9 with all other time points, except for T9 with T12. For all other time points and time lags re-test reliability was good ranging from r = 0.74 to r = 0.93. The deviation in T9 was caused by one single patient, who had a very low ASKp score at this time point (mean = 0.17) compared to the other time points (average mean = 3.11). Of note, Cronbach’s α was assessed for patients who answered all questions only. For 17 patients at least 1 question was reported as not relevant to them and we excluded them from the reliability analysis.

**Table 2:** Reliability of the ASKp

	Cronbach’s α		Retest-reliability											
			∆ 3		∆ 6		∆ 9		∆ 12		∆ 15		∆ 18	
	α	*n*	*r*	*n*	*r*	*n*	*r*	*n*	*r*	*n*	*r*	*n*	*r*	*n*
T0	0.917	38	0.656***	37	0.676***	25	0.453*	21	0.528*	20	0.494*	17	0.644**	17
T3	0.944	38	0.935***	25	0.725***	21	0.874***	20	0.830***	18	0.900***	18	–	–
T6	0.940	28	0.621**	21	0.832***	17	0.934***	15	0.909***	16	–	–	–	–
T9	0.966	30	0.806***	19	0.510	15	0.565*	18	–	–	–	–	–	–
T12	0.949	21	0.739***	19	0.867***	20	–	–	–	–	–	–	–	–
T15	0.914	20	0.964***	20	–	–	–	–	–	–	–	–	–	–
*ICC*	0.901	8	–	–	–	–	–	–	–	–	–	–	–	–
*ICC* Ɇ T9	0.961	8	–	–	–	–	–	–	–	–	–	–	–	–

*p < 0.05, **p < 0.01, ***p < 0.001

*ICC* intraclass coefficient; *ICC* *Ɇ T9* intraclass coefficient without assessment at T9, *r* Pearson correlation coefficient

### Validity: convergent and discriminant validity measure

Convergent validity was good, with a moderate correlation between the ASKp and the KPS/PSS score (r = 0.599; *p* < 0.001) (Table 3).

**Table 3:** Detailed comparison of the ASKp and KPS/PSS

	ASKp			KPS/PSS		
	*n*	*r*				
KPS/PSS	55	0.599***		*–*		
	*n*	*r*		*n*	*r*	
Age	55	0.205		55	−0.182	
Gender	*n*	*Mean (SD)*	*F*	*n*	*Mean (SD)*	*F*
Female	19	3.23(0.53)	0.653	19	9.05(0.92)	0.780
Male	36	3.07(0.77)		36	8.74(1.35)	
Adoptive Cell Therapy	*n*	*Mean (SD)*	*F*	*n*	*Mean (SD)*	*F*
No	47	3.25(0.59)	11.837***	47	9.03(0.93)	8.214**
Yes	8	2.41(0.91)		8	7.78(2.06)	
Need of devices	*n*	*Mean (SD)*	*F*	*n*	*Mean (SD)*	*F*
Yes	10	2.32(0.87)	22.298***	10	7.51(1.73)	20.132***
No	45	3.31(0.52)		45	9.15(0.84)	
Endocrine comorbidity	*n*	*Mean (SD)*	*F*	*n*	*Mean (SD)*	*F*
No	42	3.24(0.54)	4.747*	42	9.05(0.95)	5.378*
Yes	13	2.77(1.02)		13	8.19(1.73)	
GI comorbidity	*n*	*Mean (SD)*	*F*	*n*	*Mean (SD)*	*F*
No	41	3.24(0.65)	5.311*	41	9.05(0.99)	10.454**
Yes	14	2.72(0.90)		14	7.83.27(1.74)	
cGVHD global severity	*n*	*Mean (SD)*	*F*	*n*	*Mean (SD)*	*F*
No-cGVHD	36	3.26(0.53)	2.014	36	9.28(0.79)	9.721***
Moderate cGVHD	7	2.97(0.92)		7	8.50(0.86)	
Severe cGVHD	12	2.82(0.94)		12	7.76(1.71)	
No-cGVHD	36	3.26(0.53)	4.050*	36	9.28(0.79)	20.082***
Moderate & severe cGVHD	19	2.88(0.88)		19	7.91(1.49)	
Overlap cGVHD	*n*	*Mean (SD)*	*F*	*n*	*Mean (SD)*	*F*
No	46	3.23(0.64)	8.198**	46	8.97(1.04)	4.595*
Yes	9	2.54(0.074)		9	7.89(2.55)	
	*n*	*r*		*n*	*r*	
cGVHD severity 10 point scale vs NO-cGVHD	55	−0.384**		55	−0.586***	
Joint involvement	*n*	*Mean (SD)*	*F*	*n*	*Mean (SD)*	*F*
No	46	3.23(0.59)	6.130*	46	9.12(0.88)	18.246***
Yes	9	2.81(0.76)		9	7.46(1.75)	
Steroid side effects	*n*	*Mean (SD)*	*F*	*n*	*Mean (SD)*	*F*
No	46	3.28(0.53)	17.229***	46	9.09(0.86)	15.063***
Yes	9	2.28(1.14)		9	7.61(1.76)	
cGVHD-associated symptoms	*n*	*Mean (SD)*	*F*	*n*	*Mean (SD)*	*F*
No	40	3.30(0.53)	10.044**	40	9.31(0.75)	33.735***
Yes	15	2.67(0.91)		15	7.62(1.39)	

*p < 0.05, **p < 0.01, ***p < 0.001

*F*: F‑Value (explained variance/unexplained variance) of the corresponding ANOVA, *Mean (SD)*: Unstandardized Mean of ASKp respectively KPS, *r*: Pearson correlation coefficient

*ANOVA* analysis of variance, *ASKp* Activity Scale for Kids-performance, *KPS* Karnofsky Performance Scale, *PSS* Lansky Play Performance Scale

**Fig. 2:**
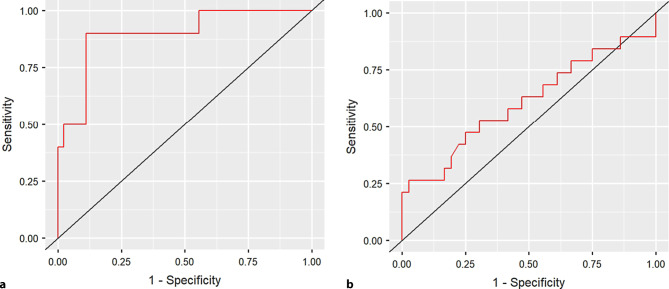
Receiver operating characteristics (ROC) curve when using ASKp scores to predict patients that need devices and patients with cGVHD. **a** Need of devices: ROC curve when using ASKp scores to predict patients thatneed devices; **b** cGVHD: ROC curve when using ASKp scores to predict patients with cGVHD

We analyzed the discriminability of the ASKp in patients who needed devices versus patients without devices. In a discriminant analysis ASKp correctly classified 90% of the patients. Of the group that needed devices 50% (*n* = 5/10) were correctly classified, whereas of the group that needed no devices 98% (*n* = 44/45) were correctly classified. Fig. 2a shows the corresponding receiver operating curve containing all possible pairs of true and false positive rates for the ASKp.

Finally, we analyzed discriminability in patients with cGVHD versus patients without cGVHD (No-cGVHD). The ASKp correctly classified 71% of patients, while 97% (*n* = 35/36) of the No-cGVHD patients were correctly assigned to their group, only 21% (*n* = 4/19) of patients with cGVHD where correctly classified (Fig. 2b).

### Ability to detect change over time

**Fig. 3:**
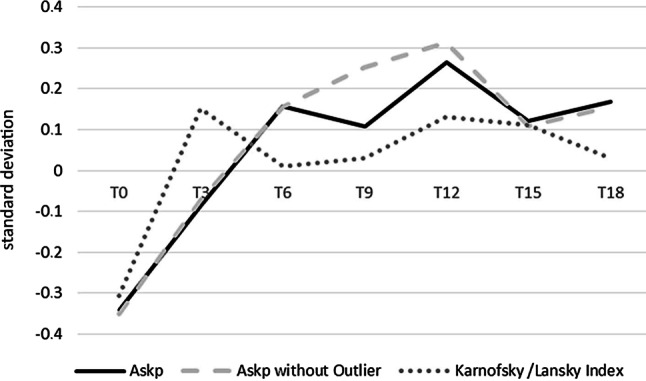
Standardized means of ASKp and KPS/PSS across time points. ASKp = M_ASK_ = *solid line*, M_ASK_ without outlier in T9 = *dashed line*, KPS/PSS = M_KPS/PSS_ = *dotted line*

To make the patient-reported ASKp and the clinician-reported KPS/PSS comparable we standardized the measurements by their average mean at every time point by the sample mean and standard deviation at this time point (M_ASK_ and M_KPS/PSS_). Fig. 3 demonstrates the development of the ASKp with and without the outlier in T9 and the KPS/PSS scores. The outlier in T9 did not affect the KPS/PSS (KPS/PSS_with outlier at T9_ = 8.85, KPS/PSS_without outlier at T9_ =8.84). Therefore, we did not include a separate calculation of the KPS/PSS scores without the outlier in T9. ASKp values increased more than a half standard deviation from T0 to T12 and the KPS/PSS scores increased nearly a half standard deviation between T0 and T3.

Accordingly, Wilcoxon sign rank test results were significant between T0 and T3 (*n* = 37, *p* = 0.001) and between T3 and T6 (*n* = 24, *p* = 0.004) for the ASKp and between T0 and T3 (*n* = 43, *p* < 0.001) for the KPS/PSS.

Furthermore, when we computed the standardized mean values across all time points (SM_ASK_) we found that the ASKp and standardized ASKp values were highly associated (r = 0.981; *p* < 0.001), even though the ASKp scores increased with time. This indicates that there are no systematic differences the groups of patients assessed at different time points. Results for both were nearly the same in all statistical analyses. Therefore, the focus will be on the further analysis of unstandardized ASKp scores.

### Detailed comparison of the patient-reported ASKp and the clinician-reported KPS/PSS

Table 3 presents ASKp and KPS/PSS scores in correlation with patients’ clinical parameters. Comparable associations for both the ASKp and the KPS/PSS were found regarding: a) no correlation with age or gender; b) a low significant correlation with patients with endocrine and GI comorbidities and c) highly significant lower scores for patients who were in the need of devices (ASKp: F = 22.298; *p* < 0.001 and KPS/PSS: F = 20.132; *p* < 0.001) and patients who suffered from steroid side effects (ASKp: F = 17.229; *p* < 0.001 and KPS/PSS: F = 15,063; *p* < 0.001). Significantly lower scores for the ASKp were found in patients after adoptive cell therapy (F = 11.837; *p* < 0.001) and in patients with overlap cGVHD (F = 8.198; *p* < 0.01) with less significant correlations in the KPS/PSS.

Similar to the discriminability analysis we found no differences between the subgroups No-cGVHD, moderate- and severe cGVHD in ASKp (F = 2.014; *p* = 0.077), whereas, differences in the KPS/PSS were highly significant (F = 9.721;* p* < 0.001). When analyzing scores of moderate and severe cGVHD patients as one group the ASKp scores showed a slightly significant correlation with cGVHD but again, a highly significant correlation in the KPS/PSS (ASKp: F = 4.050; *p* = 0.049 and KPS/PSS F = 20.082; *p* < 0.001). Moreover, only KPS/PSS scores correlated with joint and fascia manifestations of cGVHD (F = 18.246; *p* < 0.001).

### Multiple regression analyses with possible predictors of ASKp

To further evaluate the associations of clinical data with ASKp scores, we used multiple regression analyses as shown in Table 4. Considering the sample size (*n* = 55) we did not include more than five moderators at the same time into a single analysis. First, we controlled the multiple regression models for age and amount of needed help in answering the questionnaire (M1–M3). Age was not a significant predictor of ASKp. Therefore, we did not included age in further analyses. On the other side the amount of help needed in answering the questions was significantly associated with ASKp in all models no matter which moderators were included.

**Table 4:** Multiple regression analyses with possible predictors of ASKp

	M1	M2	M3	M4	M5
Age	−0.281	−0.079	0.0076	–	–
Help	−0.696***	−0.604***	−0.604***	−0.517***	−0.457***
Moderate and severe cGVHD	–	−0.319**	−0.424**	−0.092	–
Overlap cGVHD	–	–	−0.325*	−0.235*	−0.240*
Steroid side effects	–	–	–	−0.443**	–
GVHD-ASS	–	–	–	−0.251*	–
KPS/PSS	–	–	–	–	0.501***
*R*^2^	0.291***	0.400***	0.494***	0.586***	0.611***

*p < 0.05, **p < 0.01, ***p < 0.001

M1–M5: Independent multiple regression models showing standardized beta coefficient for different predictors and R2: Amount of explained variance through the model with a possible range from 0 to 1. Help: amount of needed help by answering questions of the ASKp; reference groups were patients that did not need any help answering the questions. Moderate and severe cGVHD: patients with moderate and severe cGVHD; reference group were patients with No and mild cGVHD. Overlap cGVHD: patients with overlap cGVHD: reference group were patients without overlap cGVHD. Steroid side effects: patients with side effects such as Cushing syndrome and/or myopathy; reference group were patients without steroid side effects. *GVHD-ASS: *patients with GVHD-associated symptoms; reference group were patients without GVHD associated symptoms

Interestingly, patients with moderate and severe cGVHD had lower ASKp scores compared to patients with no and mild cGVHD when the regression was controlled for amount of help needed and age (M2; β = −0.604; *p* < 0.001) as well as when overlap cGVHD was additionally included (M3; β = −0.604; *p* < 0.001), but not when overlap cGVHD, steroid side effects and cGVHD-associated symptoms were additionally included (see M4; β = 0.092; *p* = 0.505). Overlap cGVHD was negatively associated with ASKp in all models in which it was included (M3–M5). The KPS/PSS showed the highest association with ASKp (M5, β = 0.501; *p* < 0.001) besides the amount of help needed answering the questions. Both steroid side effects and cGVHD-associated symptoms were also significantly associated with ASKp score (M5, β = −0.239; *p* = 0.011; β = −0.367; *p* = 0.011).

### Associations with severity of cGVHD on a 10-point scale

Finally, we used three regression models to compare the prediction value of the ASKp and the KSP/PSS scores for cGVHD severity on a 10-point scale. Firstly, in separated simple regressions both predictors were significant. Overall, the KPS/PSS, with R2 = 0.346 (*p* <0.001), could explain more variance than the ASKp with R2 = 0.148 (*p* = 0.004). Secondly, when ASKp and KPS/PSS were included simultaneously in the model they explained less variance together R2 = 0.325 (*p* < 0.001), than the KPS/PSS alone R2 = 0.346 (*p* < 0.001). Furthermore, the ASKp failed to explain a significant amount of variance (β = 0.053; *p* = 0.709) whereas the KPS/PSS remained a significant predictor of cGVHD severity (β = −0.554; *p* <0.001).

## Discussion

Overall, the German version of the ASKp showed good psychometric properties except for ceiling effects both for less affected patients and over time. Even at study time point T9 23% scored the maximum amount of points in the ASKp and at time point T18 33% of patients scored the maximum. As stated by Klepper et al. [19] these ceiling effects may limit the usefulness for longitudinal and comparative studies including healthy control groups. Our results show that this also may apply for post-HSCT patients.

Criterion and convergent validity as well as reliability were good. One item (“I took care of my medical needs”) did not fit the scale. We assume that this is caused by the specific sample than by a translation error. Medical needs of HSCT survivors may be too complex and may vary too much between patients to fit in a physical function scale. Moreover, instruments based on self-reports are vulnerable to confounders like social desirability, cognitive development and learning effects, and other environmental and temporal influences [27]. For example, the amount of help needed answering questions was highly negatively associated with the ASKp score. Parents may compare current functioning to prior function and Hedeker et al. reported that parents rated their children’s QOL lower than the children did [2].

When we compared the patient-reported ASKp with the clinician-reported KPS/PSS regarding the association with comorbidities and steroid side effects the results were quite similar. But importantly, we did not observe a significant correlation of the ASKp with most details of cGVHD: both for severity (global severity and cGVHD severity on a 10-point scale) and joint involvement of cGVHD KPS/PSS we found a higher significant correlation with the KPS/PSS. In this regard, it seems worth considering the patient-reported KPS/PSS as an instrument to capture functional status, similar to a recent publication by Lee et al. [25].

Only differences between patients with and without overlap cGVHD were higher in ASKp than in the KPS/PSS. Specific confounders may appear in HSCT patients, which often are unable to participate in age-appropriate activities with peers. One reason for our findings could be that adolescents and children may be less aware of the implications if the clinical status is not associated with major altered functions [2].

In contrast to the HAP for adults [16] and although the ASKp was developed to assess physical disability in children with musculoskeletal impairments, our study results do not support the ASKp being appropriate to measure the impact of cGVHD severity on physical functioning so far. Consistent with the finding of another study by Christakou et al. [28] we observed that the lack of a pain scale within the ASKp is worth mentioning as many cGVHD patients especially with joint and fascia involvement and/or steroid side effects have pain along with their symptoms of cGVHD. We have previously published results from a prospective study on health-related quality of life after pediatric HSCT that most problems were detected within the domains of physical functioning and pain [12].

Of note, we observed that steroid side effects were negatively associated with the ASKp in line with findings of others that the use of steroids impact on QOL and activity scores [14].

The main limitation of this study is related to the fact that we could not conduct a linear mixed model or other more complex statistical analysis because of missing data, small sample size and, in part, non-normality of the data. Therefore, the study lacks power. The probability that even moderate effect sizes did not reach significance is rather high; however, it has been shown by other authors that the downfall of studies using patient-reported outcome measures is missing data and small sample sizes [29, 30]. Furthermore, we made multiple comparisons, which increases the possibility of random significances. Another important lesson we have learned is that the implementation of a study adjudication committee would improve the accuracy of NIH-defined cGVHD diagnosis as outlined by the study of Cuvelier et al. [30].

Apart from previously addressed problems, the recall period and the length of the self-report measure should also be considered: it may be important to use shorter recall periods with adolescents and children than with adults because this patient group may have more difficulties remembering health-related events over extended periods of time. Also, the length of the questionnaires requires careful consideration because of the wide variation in children’s ability to maintain attention to tasks. Therefore, other specific measures of physical functioning could be investigated, too. For example, we have shown in a prospective pilot study aiming to evaluate health-related QOL with the PedsQL™ and a newly developed PedsQL HSCT module to capture transplant and, specifically, cGVHD-related problems that the summary scores of the generic PedsQL and the PedsQL HSCT module showed high correlations (r = 0.89 in patients’ and r = 0.81 in parents’ assessments). Moreover, both tools discriminated between patients with and without cGVHD. The PedsQL HSCT-module was practical to use and suitable across a broad age range (2–18 years) both in patients with and without cGVHD [12]; however, it is still a pilot instrument and needs further development also regarding physical functioning and testing in a larger patient population.

## Conclusion

The ASKP patient self-report demonstrated good psychometric properties for severely impaired patients with ceiling effects in others and during the course of the study. Therefore, patients’ further progress and smaller differences may not have been detected. Moreover, the ASKp showed no significant differences for NIH-defined cGVHD severity both in global severity and on a 10-point cGVHD severity scale. In conclusion, our study was not able to prove advantages of the ASKp when compared to the KPS/PSS even though economical and patients’ effort was higher. Further prospective studies could focus on the usage of patient-reported and clinician-reported KPS/PSS in addition to physical domains of QOL instruments. Accordingly, application range of ASKp in adolescent and pediatric HSCT patients seems limited especially given the need for pediatric tools for measurement of physical functioning as an endpoint in randomized clinical trials.
