# Correction to: Psychometric properties of the Activities Scale for Kids-performance after allogeneic hematopoietic stem cell transplantation in adolescents and children: Results of a prospective study on behalf of the German-Austrian-Swiss GVHD consortium

**DOI:** 10.1007/s00508-021-01902-2

**Published:** 2021-07-01

**Authors:** Anita Lawitschka, Matthias Brunmair, Dorothea Bauer, Natalia Zubarovskaya, Rosemarie Felder-Puig, Brigitte Strahm, Peter Bader, Gabriele Strauss, Michael Albert, Irene von Luettichau, Hildegard Greinix, Daniel Wolff, Christina Peters

**Affiliations:** 1 St. Anna Children’s Hospital, SCT-Outpatient & Aftercare Clinic, Medical University Vienna and Children’s Cancer Research Institute, Kinderspitalgasse 15, 1090 Vienna, Austria; 2 Department of Psychology, University of Wuerzburg, Wuerzburg, Germany; 3 Ludwig Boltzmann Institute Health Technology Assessment, Vienna, Austria; 4 Department of Paediatrics and Adolescent Medicine, Division of Paediatric Haematology and Oncology, Medical Centre, University of Freiburg, Freiburg, Germany; 5 Division for Stem Cell Transplantation, University Children’s Hospital, Frankfurt/Main, Germany; 6 Department for Paediatric Oncology and Haematology, HELIOS Klinikum Berlin-Buch, Berlin, Germany; 7 Department of Pediatrics, Division of Pediatric Haematology and Oncology, Dr von Hauner Children’s Hospital, LMU, Munich, Germany; 8 Children’s Hospital Medical Centre, Technical University of Munich, Munich, Germany; 9 Division of Hematology, Medical University of Graz, Graz, Austria; 10 Department of Internal Medicine III, University Hospital Regensburg, F. J. Strauss Allee 11, 93053 Regensburg, Germany

**Correction to:**

**Wien Klin Wochenschr 2020**

10.1007/s00508-020-01641-w

The original version of this article unfortunately contained a mistake. Due to a breach of copyright, Fig. 1 “ASK©-Performance SCORE SHEET. The ASKp domains, items and response option” had to be removed from the article. The authors apologize for the mistake. The original article has been corrected.

